# Could Proteomic Research Deliver the Next Generation of Treatments for Pneumococcal Meningitis?

**DOI:** 10.1155/2009/214216

**Published:** 2009-05-27

**Authors:** U. R. Goonetilleke, S. A. Ward, S. B. Gordon

**Affiliations:** ^1^Molecular and Biochemical Parasitology, Liverpool School of Tropical Medicine, Pembroke Place, Liverpool L3 5QA, UK; ^2^Pulmonary Immunology, Liverpool School of Tropical Medicine, Pembroke Place, Liverpool L3 5QA, UK

## Abstract

*Streptococcus pneumoniae* is the most common bacterial cause of community-acquired meningitis worldwide. Despite optimal antibiotic therapy and supportive care, the mortality of this condition remains very high at 20–30% in the developed world and over 60% in under-resourced hospitals. In developed countries, approximately half of the survivors suffer intellectual impairment, hearing loss, or other neurological damage. There is an urgent need for more information about the mechanisms of brain damage and death in pneumococcal meningitis so that new treatments can be designed. Using proteomic techniques and bioinformatics, the protein content of cerebrospinal fluid can be examined in great detail. Animal models have added greatly to our knowledge of possible mechanisms and shown that hippocampal apoptosis and cortical necrosis are distinct mechanisms of neuronal death. The contribution of these pathways to human disease is unknown. Using proteomic techniques, neuronal death pathways could be described in CSF samples. This information could lead to the design of novel therapies to minimize brain damage and lower mortality. This minireview will summarize the known pathogenesis of meningitis, and current gaps in knowledge, that could be filled by proteomic analysis.

## 1. Clinical Problem of Meningitis

**Figure 1:**
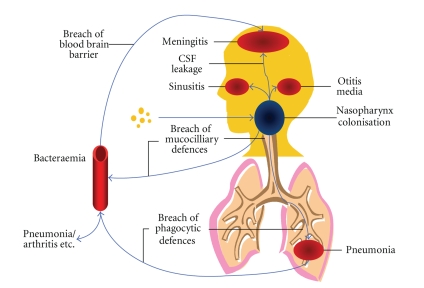
When pneumococci spread to the sinuses, ear, lung, and blood stream, diseases such as sinusitis, otitis media, pneumonia, and septicaemia can result. Invasion of the central nervous system (CNS) by colonising pneumococci follows an alteration in the balance between the virulence of the bacteria and the defences of the patient. Factors such as common colds or other upper respiratory virus infections alter the lining of the respiratory tract and allow bacteria to enter the bloodstream. Pneumococci then actively translocate across intact endothelial layers by means of specific receptor binding and translocation. Endothelial cells normally separate the blood from neuronal tissue forming a protective blood-brain barrier (BBB). The integrity of the BBB is compromised by apoptosis of endothelial cells. The BBB breakdown allows further invasion of cerebrospinal fluid (CSF).

Infection of the membranes surrounding the central nervous system (meninges) results in meningitis. *Streptococcus pneumoniae*, an ovoid gram-positive bacterium [1], is the most common cause of bacterial meningitis [2]. Pneumococci are able to colonise the nasopharynx without developing any serious consequences. Pneumococcal carriage rates in young children vary from over 40% in developed nations such as USA to 87% in developing nations such as Gambia [3, 4]. Pneumococcal carriage rates in adults vary from approximately 10% in developed nations such as USA to 51% in developing nations such as Gambia [5]. When pneumococci spread to the sinuses, ear, lung, and blood stream, diseases such as sinusitis, otitis media, pneumonia, and septicaemia can result (Figure 1). As an example *pneumococcal* meningitis in Malawi has a high fatality rate of 65% [6] and survivors may develop long-term neurological sequelae, including hearing loss and other focal neurological deficits [7]. 

## 2. Pathogenesis of Meningitis

Invasion of the central nervous system (CNS) by colonising pneumococci follows an alteration in the balance between the virulence of the bacteria and the defences of the patient. Factors such as common colds or other upper respiratory virus infections alter the lining of the respiratory tract and allow bacteria to enter the bloodstream. Pneumococci then actively translocate across intact endothelial layers [8] by means of specific receptor binding and translocation. Endothelial cells normally separate the blood from neuronal tissue forming a protective blood-brain barrier (BBB). The integrity of the BBB is compromised by apoptosis of endothelial cells. The BBB breakdown allows further invasion of cerebrospinal fluid (CSF) [9–11]. It has been observed in some children that bacteria can translocate directly from the nasopharynx into the CNS via olfactory neurones [12]. A nonhaematogenous route has also been demonstrated in animal models [13].

The host inflammatory response to the pneumococcus is initiated by pneumococcal toxins such as pneumolysin and hydrogen peroxide [14, 15]. Most of the tissue damage associated with meningitis is caused by host responses including the action of phagocytes, secreted granular toxins, cytokines and leukotrienes, matrix metalloproteinases, and the direct pressure effect of cerebral oedema causing ischaemia [16]. In addition pneumococcal proteins have been shown to contribute to neuronal cell death in animal models [17]. Neuronal cell death has been determined to occur via three distinct pathways [18] which are illustrated in Figure 3.

- Classic caspase-3-dependent cell death which leads to apoptosis or programmed cell death.
- Caspase-3-independent cell death which leads to pyknosis (irreversible condensation of chromatin in the nucleus of a cell undergoing programmed cell death or apoptosis).
- Necrosis, the unnatural death of cells and living tissue through cell swelling, chromatin digestion, and disruption of the plasma membrane and organelles.

## 3. Neuronal Cell Death in the Hippocampus

**Figure 2:**
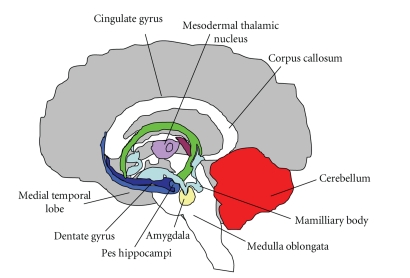
The hippocampus is a part of the forebrain, located in the medial temporal lobe. The hippocampus is used in storing and processing spatial information. In the rabbit model of pneumococcal meningitis, hippocampal apoptosis was found to be the predominant form of neuronal damage. In humans, apoptosis and pyknosis have been identified in the dentate gyrus of the hippocampus. Apoptosis primarily affects the subgranular zone containing recently divided immature neurons. Pyknosis occurs throughout the dentate granular cell layer.

Animal models have been used to determine the mechanism of pneumococcal related neuronal apoptosis. In the rabbit model of pneumococcal meningitis, hippocampal apoptosis was found to be the predominant form of neuronal damage [19, 20]. Inhibition of phosphorylcholine synthesis in mitochondria of neurons in the hippocampal dentate gyrus leads to mitochondrial release of apoptosis inducing factor (AIF) which in turn causes pyknosis of the hippocampus. In an adult mouse model both caspase-dependent and independent forms of neuronal cell death have been described in the dentate gyrus of adult mice [21]. Bacterial cell wall products initiate mitochondrial release of cytochrome c leading to classic toll-like receptor (TLR) dependent-caspase-3 mediated apoptosis occurring more widely in the brain. Infant rat models of pneumococcal meningitis showed similar neuronal damage patterns to human disease [22, 23]. In the infant rat meningitis model, apoptosis and pyknosis of neurons have been identified in the dentate gyrus of the hippocampus (Figure 2). In humans apoptosis has been identified in the dentate gyrus [24]. Apoptosis primarily affects the subgranular zone containing recently divided immature neurons. Pyknosis occurs throughout the dentate granular cell layer. Both mature and immature neurons are affected as a result [20].

## 4. Neuronal Cell Death in the Cortex by Necrosis

**Figure 3:**
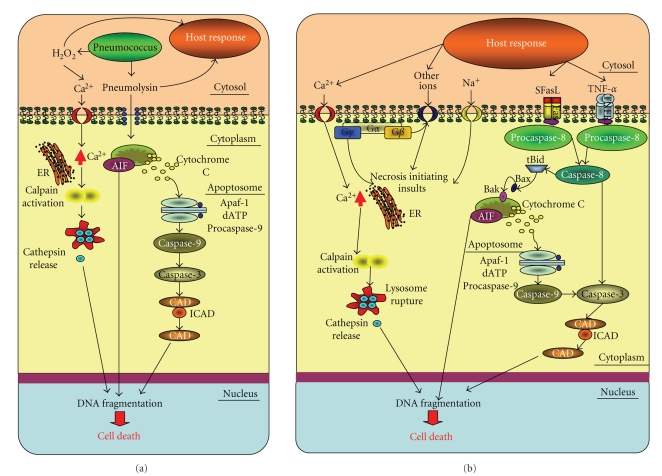
(a) The cell wall of *S. pneumoniae* has a diverse protein population. Proteins such as pneumolysin can trigger apoptosis on entering cells by destruction of the mitochondria. In addition oxidising components such as hydrogen peroxide can trigger apoptosis and necrosis. (b) The host immune response will most likely be made up of complement and cytokines which can activate transmembrane death receptors such as Fas. This will cause receptors to aggregate together on the cell surface leading to apoptosis. The adaptor protein Fas-associated death domain protein (FADD) activates caspase-8, an initiator protein, to form a signal complex to directly activate caspase-3. Active caspase-8 can also cleave BID protein to tBID, which acts as a signal on the membrane of mitochondria to facilitate the release of cytochrome c in the intrinsic pathway. The mitochondrial stress pathway is initiated when proapoptotic proteins in the cytoplasm, BAX, and BID stimulate the rupture of the mitochondria. The release of mitochondrial content is aided by the protein BAK. In the caspase dependant pathway, cytochrome c released from the mitochondria forms a complex in the cytoplasm with adenosine triphosphate (ATP) and apoptotic protease activating factor-1 (Apaf-1). This complex activates caspase-9, an initiator protein. In return, the activated caspase-9 works together with the complex of cytochrome c, ATP, and Apaf-1 to form an apoptosome, which in turn activates caspase-3, the effector protein that initiates degradation. The caspase independent pathway (pyknosis) is as a result of apoptosis inducing factor (AIF). The necrotic pathway is activated in severe meningitis. Alterations in the concentration of cytoplasmic calcium could signal the mobilisation of executioner cathepsin proteases and other hydrolases, through calpain activation. Calpains have been implicated in the activation of proapoptotic caspase proteases; hence the later steps of necrosis correlate with the later steps of apoptosis.

A feature of severe pneumococcal meningitis is ischaemic damage of neurons in the ischaemic core of the cortex which results in necrosis in addition to caspase-3 dependent cell death in the ischemic core and penumbra [22, 25, 26]. Inflammation of the meninges leads to oxygen and glucose deprivation of neuronal cells. The release of excitatory neurotransmitters from glutamatergic neurons leads to glutamate receptor overactivation, Ca^2+^ influx and subsequent injury, and eventually neuronal necrosis [27]. Neuronal necrosis and neuronal apoptosis may share a final common path [28], that is, the mitochondrial pathway as illustrated in Figure 3.

## 5. Steroid Therapy

Most of the information that is known about inflammation has come from animal models, in which neuronal injury may be reduced by modulation of the inflammatory response with steroid adjuvant therapy [29]. In developed nations, steroid adjuvant therapy has been shown to reduce deaths in some adults, particularly in patients with mild pneumococcal meningitis [30]. On the other hand, a large paediatric trial in Malawi demonstrated no benefit from steroids in children with bacterial meningitis [31]. Further, a double blind, randomised, placebo controlled trial of dexamethasone adjuvant therapy in adults with bacterial meningitis in Malawi also showed no advantage at 40 days [32]. The difference seen between Europe and Malawi is likely to have resulted from differences in the severity of the cases. There remains an urgent need for novel adjuvant therapy in the treatment on pneumococcal meningitis worldwide.

## 6. Potential for New Therapy

Critical pathways for new therapy should clearly target the apoptotic and necrotic pathways. For example, citicholine is an intermediate in the synthesis of phosphorylcholine in mitochondrial and cell membranes. It has been shown to prevent neuronal damage when given before and after bacterial infection in animal models of meningitis, regardless of the route of infection [15, 33]. Alternatively matrix metalloproteinase inhibitors could prevent blood brain barrier damage. Matrix metalloproteinase (MMPs) are a family of zinc-dependent endopeptidases that show affinity to different components of the extracellular matrix. They have been shown to play a role in the breakdown of the blood-brain barrier and the facilitation of neuroinflammation in bacterial meningitis [34]. In bacterial meningitis, MMPs may contribute to the development of brain injury by both their proteolytic activity on the extracellular matrix and their ability to increase the levels of soluble TNF-*α*, a pivotal element in the meningeal inflammatory process. TNF-*α* is a strong stimulus for the release and activation of MMPs in the brain [35, 36].

## 7. Critical Gaps in Knowledge

There are critical gaps in knowledge that need to be addressed before new therapies can be implemented in meningitis. In particular there is insufficient data to link human death in meningitis with the mechanisms observed in animal models. High levels of CSF apoptosis proteins in patients with neurological damage or death would provide a basis for trials of citicholine. Alternatively, high levels of MMP and MMP-related damage would provide a case for the use of MMP inhibitors. Proteomics' methods provide a modern means of obtaining these pivotal data. Proteomics is the qualitative and quantitative analysis of all expressed proteins present in cells, tissues, or organisms at certain time and under different conditions [37]. 

The application of proteomics to CSF samples and serum will allow the presence or absence of both high and low abundant proteins to be associated with neurological damage and death in meningitis. Proteomics' approaches allow the analysis of a large spectrum of host and pathogen proteins but cannot yet be applied to the bacterial cell wall components such as lipoteichioic acid (LTA) of pneumococcus. These methods have already been applied in malaria and tuberculosis but have not yet advanced to the treatment of pneumococcal meningitis [38, 39].

## 8. Proteomic Methods That Can Be Applied to CSF Analysis

**Figure 4:**
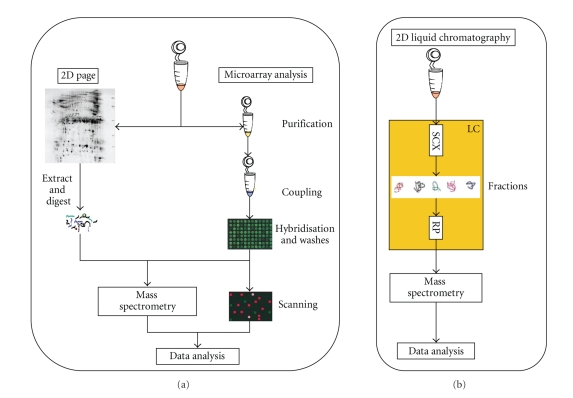
(a) Proteins from mixtures are analysed using 2D PAGE followed by mass spectrometry. The identification of proteins is from the spectra produced from the peptides of the digested proteins which provide a unique fingerprint from the peptides. Protein microarrays consist of different protein binding molecules, for example, antibodies at separate locations on a chip made from glass or silicone to capture molecules to detect proteins from cell lysate solutions. The proteins can be visualised using a fluorometric or colorimetric analysis or the spots can be analysed by mass spectrometry. (b) Multidimensional liquid chromatography mass spectrometry (LC-MS) involves digestion of a complex protein sample initially using a strong cation exchange (SCX) column. The resulting peptides are separated by LC into fractions. The resulting fractions are separated further using a reversed-phase (RP) column and characterized by mass spectrometry using shotgun sequencing of the peptides.

A basic proteomic approach to meningitis will involve the comparison of protein expression in normal and disease CSF to identify aberrantly expressed proteins [37]. Proteomic techniques applicable to CSF are either top-down, that is, a “shotgun” approach to protein identification or bottom-up, that is, the identification of proteins from the peptide spectra of a digested protein [40, 41] as shown in Figure 4.

## 9. Top-Down Proteomics

2-Dimensional Polyacrylamide Gel Electrophoresis (2D PAGE) with protein identification using mass spectrometry [42] involves the separation of proteins according to isoelectric point and molecular weight. The proteins are excised out of the gels and either directly interrogated by mass spectrometry (true top down proteomics) or are digested with specific enzymes prior to analysis using mass spectrometry. The isoelectric point of a protein and their mass aids identification of proteins and posttranslational modified proteins can be identified through the position in the gel at a comparatively low cost. However 2D PAGE is slow, and high-abundant proteins in the gel can confound small proteins.

Protein microarrays consist of different protein binding molecules, for example, antibodies spotted at separate identifiable locations on a chip made from glass or silicone to detect proteins from cell lysate solutions [43]. This technique has high sensitivity and can simultaneously analyse thousands of proteins within a single experiment. Antibody specific profiling allows analysis of complex protein mixtures; however this limits the technique to the detection of specific targets and does not give a complete view of the proteins involved in a process.

## 10. Bottom-Up Approaches

Multidimensional liquid chromatography mass spectrometry (LC-MS) involves digestion of a complex protein sample. The resulting peptides are separated by chromatography into fractions. The resulting fractions are separated further by chromatography and characterized by mass spectrometry using shotgun sequencing of the peptides, that is, the peptide fractions are reassembled to give the overall sequence to generate protein lists. This technique is almost entirely automated and has increased sensitivity to enhance the detection of low-abundance proteins. It has a greater success in the identification of proteins in very complex mixtures [42, 44, 45]. However, analysis times can be long, and the resulting data is complex. Also high abundance proteins tend to confound smaller proteins in the analysis.

## 11. Western Blotting

An alternative protein analysis technique is western blotting in which proteins in CSF can be confirmed using specific antibodies. This technique allows specific targets to be compared between sample groups. It relies on previous identification of proteins of interest.

## 12. Nonprotein Techniques

Glycan structures present on the cell wall such as lipoteichioic acid (LTA) and peptidoglycan (PG) play an important role in inflammation but are not accessible to proteomic approaches. Glycans can be measured using specialist techniques for example the silkworm larvae (SLP) test was developed to analyse PG in CSF [46]. Similarly LTA can be measured using an enzyme immunoassay [47].

## 13. Host and Pathogen Proteins

Proteomic comparisons of infected and normal CSF can be expected to differ in the concentration of both pneumococcal proteins and host proteins. Identification of pneumococcal proteins associated with poor outcome may suggest either therapeutic possibilities or vaccine candidates. Host proteins associated with poor outcome may suggest pathways amenable to immunomodulation or therapeutic intervention.

## 14. Pneumococcal Proteins

The cell wall of *S. pneumoniae* has a diverse protein population, and pathogenic expression of pneumoccal proteins is associated with adherence to and colonisation of mucosal surfaces, resistance to specific and nonspecific host defences, penetration, and invasion of host tissues, and generation of tissue damage mediated either directly by toxins or indirectly via inflammatory responses as summarised in Table 1. All the proteins listed in Table 1 have been described in experimental studies including animal models of meningitis and have been found to exhibit an effect on inflammation, and toxicity for example *N*-acetylmuramoyl-L-alanine-amidase (LytA) is an autolytic enzyme required during cell division. Its role in pneumococcal meningitis is unknown but has been shown in various animal models to mediate toxicity and inflammation [48]. Proteins such as pneumolysin can stimulate the host response and also enter cells though pore formation. It has the ability to trigger apoptosis on entering cells by destruction of the mitochondria [48]. In addition oxidising components such as hydrogen peroxide can also trigger apoptosis and necrosis. Both pneumolysin and autolysin have been shown to play a crucial role in the pathogenesis of pneumococcal meningitis in an adult rat model of meningitis [69]. Neuraminidases are a group of enzymes which can cleave terminal sialic acid residues from a wide variety of glycan structures [55]. The pneumococcus produces two distinct neuraminidases, *N*-acetylneuraminic acid (NanA) and *endo*-*β*-1, 4-*N*-acetylglucosaminidase (NanB) [70, 71]. There are several conflicting publications on the precise role of NanA in pneumococcal disease; however an Otitis media chinchilla model that revealed that NanA-deficient pneumococci are significantly less able to colonise and persist in the nasopharynx and middle ear than NanA-sufficient wild-type pneumococci [56]. The relative contribution of NanB to disease has not been reported in either a sepsis or pneumonia model.

## 15. Host Proteins

The host immune response will most likely make up the majority of proteins present in the CSF because these proteins will include host immune response factors such as complement and cytokines as well as specific immunoglobulins and proteins from serum leaking to the CSF as a result of the blood brain barrier breakdown (Table 2). These will include signalling molecules such as tumour necrosis factor alpha (TNF-*α* [72], Fas and Fas-associated death domain (FADD) protein, (Table 3) [68]. These proteins can lead to apoptosis through activation of transmembrane death receptors, such as Fas which causes receptors to aggregate together on the cell surface. This activates the adaptor protein Fas-associated death domain (FADD) protein, which in turn activates caspase-8, an initiator protein, to form a signal complex. This complex is now able to directly activate caspase-3, an effector protein, to initiate degradation of the cell. Active caspase-8 can also cleave BID protein to tBID, which acts as a signal on the membrane of mitochondria to facilitate the release of cytochrome c in the intrinsic pathway. The mitochondrial stress pathway is initiated when a stress signal is activated, proapoptotic proteins in the cytoplasm, BAX, and BID stimulate the rupture of the mitochondria. The release of mitochondrial content is aided by the protein BAK. In the caspase dependant pathway, cytochrome c released from the mitochondria forms a complex in the cytoplasm with adenosine triphosphate (ATP) and apoptotic protease activating factor-1 (Apaf-1). This complex activates caspase-9, an initiator protein. In return, the activated caspase-9 works together with the complex of cytochrome c, ATP, and Apaf-1 to form an apoptosome, which in turn activates caspase-3, the effector protein that initiates degradation. The caspase independent pathway (pyknosis) is a result of apoptosis inducing factor (AIF). The necrotic pathway is activated in severe meningitis. Alterations in the concentration of cytoplasmic calcium signal the mobilisation of executioner cathepsin proteases and other hydrolases, through calpain activation. Calpains have been implicated in the activation of proapoptotic caspase proteases; hence the later steps of necrosis correlate with the later steps of apoptosis. 

Proteomic analysis of CSF will allow dominant pathways to be determined and the relative importance of apoptosis and necrosis to be estimated in patients and neurological damage.

## 16. Conclusion

A novel therapy is needed to improve outcome in meningitis. Animal models that suggest mechanism of neuronal injury are amenable to therapy. Critical information is still needed to move from animal models into human trials. This pivotal information could be provided by proteomic analysis of CSF.

## Figures and Tables

**Table 1:** The cell wall of *S. pneumoniae* has a diverse protein population, and pathogenic expression of pneumoccal proteins are associated with adherence to and colonisation of mucosal surfaces, resistance to specific and nonspecific host defences, penetration and invasion of host tissues, and generation of tissue damage mediated either directly by toxins or indirectly via inflammatory responses.

Protein	Description	Action	References
LytA	Enzyme required during cell division	Hydrolyses amide bonds between muramic acid and L-Alanine residues	[48, 49]
PspA	Ranges from 67–99 kDa in size. Anchored to the outer layer of the plasma membrane	Reduces complement mediated clearance and phagocytosis of *S. pneumoniae*. Inhibits complement activation, thereby limiting opsonisation of pathogens by complement protein 3 (C3)	[50]
Pneumococcal histidine triad (Pht)	Novel family of cell surface-exposed pneumococcal proteins	Consists of PhtA, PhtB, PhtD, and PhtE. PhtB and PhtE. Induces antibodies capable of protecting mice against pneumococcal sepsis and death	[51, 52]
PspC	Surface protein choline binding domain has 90% homology to PspA	Binds to the polymeric immunoglobulin receptor and mediates invasion across human nasopharyngeal epithelial cells	[53, 54]
Neuraminidases, for example, NanA and NanB	Cleaves terminal sialic acid residues from a wide variety of glycolipids, glycoproteins, and oligosaccharides	The precise role of NanA in pneumococcal disease is unknown. The relative contribution of NanB to disease has not been reported in either a sepsis or pneumonia model	[55, 56]
Heat shock proteins	A highly conserved set of proteins	Heat stress proteins are produced after penetration from the nasal mucosa (30 to 34°C) into the blood and/or meninges (37°C)	[39]
Hyaluronate lyase (Hyal)	Covalently linked to the cross-bridges of the cell wall peptidoglycan	Degrades essential components of the host's extracellular matrix (ECM), hyaluronan (HA), unsulfated chondroitin (CH), and certain chondroitin sulfates (CHSs)	[57]
Pneumococcal surface antigen A (PsaA)	34.5 kDa protein covalently anchored to the cell membrane	Belongs to an ATP binding cassette-(ABC-) type transport system and constitutes the extracellular component responsible for solute (metal) binding	[58]
Pneumolysin (Hemolysin or Ply)	53-kDa protein	Binds to membrane cholesterol and inserts the toxin into the lipid bilayer. Induces leakage of solutes	[59]
Penicillin-binding proteins (PBPs)	*S. pneumoniae* carry a relatively simple set of six PBPs	Catalyse the polymerisation of glycan chains and transpeptidation of pentapeptidic moieties within the structure of the peptidoglycan	[60]
Pneumococcal iron uptake (Piu) and iron acquisition (Pia)	Lipoprotein components of iron ABC transport systems	Essential for iron uptake. Pia is the dominant iron transporter. PiuA and PiaA have been shown to be present in all pneumococcal species	[61]

**Table 2:** The host immune response will most likely make up the majority of proteins present in the CSF because these proteins will include host immune response factors such as complement and cytokines as well as specific immunoglobulins and proteins from serum leaking to the CSF as a result of the blood brain barrier breakdown.

Protein	Description	Action	References
Complement components, for example, C3b, iC3b, or C4b (CR1, CR3)	Consists of a number of small proteins found in the blood, normally circulating as inactive zymogens	Help to clear pathogens from an organism	[62]
IL-6	A proinflammatory cytokine	Secreted by T cells and macrophages to stimulate immune response to trauma, leading to inflammation	[63]
Interleukin-1 (IL-1)	A superfamily consisiting of IL-1*α*, IL-1*β*, and the IL-1 receptor antagonist (IL-1RA)	They control lymphocytes. IL-1*α* and IL-1*β* are produced by macrophages, monocytes, and dendritic cells	[64]
IgG	The most abundant immunoglobulin. Equally distributed in blood and in tissue liquids	Activates complement (classic pathway), opsonization for phagocytosis, and neutralisation of their toxins	[65]

**Table 3:** Poteins associated with the apoptotic pathway could potentially be discovered in the CSF after cell death. The levels of these proteins can be expected to increase during pneumococcal meningitis as a result of both the inflammatory response and the release of pneumococcal proteins.

Protein	Description	Action	References
Cytochrome C (Cyt C)	A small heme protein found loosely associated with the inner membrane of the mitochondrion	Cause ER calcium release. The overall increase in calcium triggers a massive release of additional cyt c, which then acts in the positive feedback loop to maintain ER calcium release through the inositol 3 phosphate receptors. This release in turn activates caspase-9	[66]
Tumour necrosis factor (TNF-*α*)	TNF acts via the TNF receptor (TNF-R) and is part of the extrinsic pathway for triggering apoptosis	TNF-R associates with procaspases through adapter proteins (FADD, TRADD, etc.)	[67]
Caspases	Proteases, which exist as inactive proenzymes	Play essential roles in apoptosis (programmed cell death) and inflammation	[10]
Fas	Ligand which associated with the forms the Death Inducing Signalling Complex (DISC) upon ligand binding	Fas pathway is sufficient to induce complete apoptosis in certain cell types through DISC assembly and subsequent caspase-8 activation	[68]
Fas-associated death domain protein (FADD)	An adaptor molecule that bridges the Fas-receptor, and other death receptors, to caspase-8 through its *death domain*	Forms the death inducing signalling complex (DISC) during apoptosis	[68]
BAX	A proapoptotic member of the Bcl-2 protein family	Activated Bax forms an oligomeric pore in the outer membrane	[10]
Apoptosis inducing factor (AIF)	A flavoprotein found in the mitochondrial intermembrane space in healthy cells	Essential for nuclear disassembly in apoptotic cells	[10]
